# Hepatocellular Carcinoma cells: activity of Amygdalin and Sorafenib in Targeting AMPK /mTOR and BCL-2 for anti-angiogenesis and apoptosis cell death

**DOI:** 10.1186/s12906-023-04142-1

**Published:** 2023-09-19

**Authors:** Tarek El-Sewedy, Afrah Fatthi Salama, Amro E. Mohamed, Nashwa M. Elbaioumy, Ali H. El-Far, Aisha Nawaf Albalawi, Alaa Elmetwalli

**Affiliations:** 1 Department of Applied Medical Chemistry, Medical Research Institute, Alexandria University, Alexandria, Egypt; 2 Biochemistry Section, Chemistry Department, Faculty of Science, Tanta University, Tanta, 31527 Egypt; 3 Department of Biochemistry, Faculty of Veterinary Medicine, Damanhour University, Damanhour, 22511 Egypt; 4 Biology Department, University College of Haqel, University of Tabuk, Tabuk, KSA Saudi Arabia; 5 Department of Clinical Trial Research Unit and Drug Discovery, Egyptian Liver Research Institute and Hospital (ELRIAH), Mansoura, Egypt

**Keywords:** HCC, AMPK /mTOR, Angiogenesis, Sorafenib, Molecular docking, Amygdalin

## Abstract

**Background:**

Sorafenib (Sor) is the only approved multikinase inhibitor indicated for the treatment of HCC. Previous studies have shown that amygdalin (Amy) possesses anticancer activities against several cancer cell lines; we suggested that these compounds might disrupt AMPK/mTOR and BCL-2. Therefore, the current study used integrated in vitro and *in silico* approaches to figure out Amy and Sor’s possible synergistic activity in targeting AMPK/mTOR and BCL-2 for anti-angiogenesis and apoptosis cell death in HepG2 cells.

**Results:**

Notably, Amy demonstrated exceptional cytotoxic selectivity against HepG2 cells in comparison to normal WI-38 cells (IC_50_ = 5.21 mg/ml; 141.25 mg/ml), respectively. In contrast, WI-38 cells were far more sensitive to the toxicity of Sor. A substantial synergistic interaction between Amy and Sor was observed (CI_50_ = 0.56), which was connected to cell cycle arrest at the S and G2/M stages and increased apoptosis and potential necroptosis. Amy and Sor cotreatment resulted in the highest glutathione levels and induction of pro-autophagic genes AMPK, HGMB1, ATG5, Beclin 1, and LC3, suppressed the mTOR and BCL2 anti-apoptotic gene. Finally, the docking studies proposed that Amy binds to the active site of the AMPK enzyme, thus inhibiting its activity. This inhibition of AMPK ultimately leads to inhibition of mTOR and thus induces apoptosis in the HepG2 cells.

**Conclusion:**

Although more in vivo research using animal models is needed to confirm the findings, our findings contribute to the evidence supporting Amy’s potential anticancer effectiveness as an alternative therapeutic option for HCC.

**Supplementary Information:**

The online version contains supplementary material available at 10.1186/s12906-023-04142-1.

## Background

The most common malignant tumor, hepatocellular carcinoma (HCC), arises from well-differentiated hepatocytes [1]. It is the most common type of malignant tumor observed worldwide, and it usually results in death within 6–20 months [2, 3]. Biological occurrences connected to tumor development are among HCC’s distinguishing characteristics [4]. The distinguishing characteristics include, in brief, persistent proliferative signaling, evasion of growth suppressors, prevention of cell death, the establishment of replicative immortality, induction of angiogenesis, and activation of invasion and metastasis [5, 6]. The microenvironment of the cell and the interactions of many growth factors have a role in tumor development and survival [2, 7]. The most recent data on cancer stem cells (CSC) revealed, however, that liver cancer is linked to defective signaling pathways and its receptors, including transforming growth factor (TGF) [8], vascular endothelial growth factor, fibroblast growth factor, Wnt/mitogen-activated protein kinases, phosphoinositide 3-kinase, mechanistic target of rapamycin, and Hedgehog pathways [9].

Targeting malignant disorders by attacking protein kinases has become more popular recently [10]. A member of the family of PI3Ks/mTOR is a physiologically stable serine/threonine protein kinase [11]. It performs a variety of functions, including regulating metabolism, aging, and cell proliferation [12]. Solid tumors, including HCC, have been related to mTOR up-regulation, which is typically associated with a poor prognosis and fast recurrence [13, 14]. Furthermore, genomic investigations showed that the proteins PI3K and AKT, which are engaged in the mTOR pathway’s upstream signaling, are important actors in the unregulated network signaling pathways in HCC [15–17]. As a result, rapamycin/rapalogs and several mTOR second-generation blockers have been developed and are now being evaluated in clinical studies for the treatment of HCC [18, 19].

In this context, several members of our group have been trying to understand molecular pathogenesis, leading to improvements in the treatment of HCC. Radical liver removal, orthotropic liver transplantation, and locoregional therapy make up the main therapeutic modalities for HCC [20, 21]. However, systemic treatment, which includes molecular targeted therapy, nutraceuticals, and chemoprevention, is the only option for uncontrolled HCC. Sorafenib (Sor) can block receptor tyrosine kinases, which are involved in the development of new blood vessels, as well as cell proliferation as a result [22]. Sor has been shown in preclinical studies to block several receptors, including VEGFR13, PDGFR, cKIT, and FLT3 [23, 24]. However, the side effects of Sor treatment also include symptoms that are frequently brought on by drugs, such as hypertension, asthma, and esophageal irritation [25, 26].

Furthermore, bevacizumab and doxorubicin have not been coupled with any other standardized drugs [27]; only Sor has been done [28]. Sor combination therapy may therefore be more effective than using different chemotherapeutic drugs alone [29]. In that regard, several phytochemical components likewise share chemopreventive and angiopreventive qualities and ought to be tested in combination with chemotherapeutic substances to lessen drug-induced toxicity [30, 31].

Amygdalin (Amy) is a naturally occurring cyanogenic glycoside that may be found in a variety of fruits, such as the kernels of apricots, peaches, and bitter almonds [32]. Amy is not toxic by itself, but the β-glucosidase enzyme decomposes it into hydrocyanic acid (HCN), which stimulates the lysosome enzymes and raises the acidity of cancer cells, causing them to lyse [33]. β-glucosidase shows 1000–3000 times higher activity in tumor cells than in normal cells due to the presence of lactate generated during cancer cell anaerobic respiration [34]. HCN can also kill cancer cells by elevating the acidic content of the cell leading the lysosome to release its enzymatic content, causing the cells to lyse [35]. Moreover, detoxification of HCN to thiocyanate requires the mitochondrial enzyme rhodanese, which is more active in normal tissues but has lower activity in cancer cells. Thus, a combination of abundant cyanide liberating b-glucosidase activity together with a deficiency of the cyanide detoxifying rhodanese activity could provide a selective advantage for the killing of cancer cells by amygdalin without having plentiful harmful effects on normal cells [36]. Additionally, Amy prevents metastasis and inhibits mitochondrial cytochrome C oxidase [37]. It can also affect other signaling pathways [38]. Therefore, the unique combination of Sor and Amy-based anti-cancer activity may be a superior choice for HCC-targeted treatment [39].

Here, this study aimed to test the innovative activity of Amy and/or Sor against HCC using the HepG2 cell line. We suggested that these compounds might disrupt the prototypical survival pathway known as AMPK/mTOR and BCL-2, which is becoming more and more associated with the development of HCC carcinogenesis [38, 40]; additionally, we assessed the simulated binding process toward AMP-activated protein kinase (AMPK)/mTOR and B-cell lymphoma 2 (BCL-2) as the molecular target for inhibition using molecular docking, and we employed quantitative PCR to analyze the genes expression.

## Methods

### Chemicals and reagents

Amy was purchased from Sigma Aldrich, USA ( CAS-No. 29883-15-6), Sorafenib Tosylate 200 mg from Cipla, India, and dimethyl sulfoxide (DMSO) from Sigma–Aldrich ( CAS -No. 67-68-5), USA, 3-(4,5-dimethylthiazoil-2-yl)-2,5 diphenyltetrazolium bromide [MTT] from Sigma–Aldrich, USA (CAS-No. 298-93-1), Propidium iodide (CAS-No. 25535-16-4), Ethidium bromide (CAS-No. 1239-45-8), Acridine orange CAS-No. 65-61-2), and DAPI (CAS-No. 28718-90-3) were procured from Sigma Chemicals Co. (St. Louis, MO, USA). All other analytical grade chemicals used in the study were obtained from Biomed laboratories, Egypt. Stock solutions of Sor were freshly prepared in DMSO and Amy in DMEM media. Human hepatocellular carcinoma (HepG2) and normal lung WI-38 human cell lines were obtained from VACSERA, Dokki-Giza, Egypt.

### Cell Culture and treatment

The HepG2 and normal lung WI-38 human cell lines were cultured in DMEM medium (Lonza, BioWhittaker®, USA ( CAS-No. 12–614) supplemented with 10% fetal bovine serum (FBS) (Sigma, USA) (CAS-No. 1943609-65-1), and 100 µg/ml penicillin, 100 µg/ml streptomycin (Lonza, BioWhittaker®, USA)( Cat. No. DE17-602E), at 37 °C in a humidified incubator under 5% CO_2_. Cells were subcultured when reaching 80%-90 confluency and were split in a 1:6 ratio before treatments that were routinely performed on 40–50% confluent cells.

Except for the morphology analysis that was assessed for 24 and 48 h, all treatments were performed for 48 h using the predetermined half IC_50_ dose (IC_50/2_) of the drugs, Amy was used at 2.6 mg/ml against HepG2 and 70.6 mg/ml against WI-38 cells. On the other hand, Sor 1.1 µM and 0.29 µM doses were used against HepG2 and WI-38 cells, respectively. Finally, for the combination treatments, 2.03629 mg/ml Amy and 0.40726 µM Sor were used. Cell culture experiments were performed in triplicates, and the results of three independent experiments were used for statistical analysis.

### Cell viability assay

The effect of Amy & Sor on the viability of HepG2 and WI-38 cells was determined by the MTT colorimetric assay kit (Sigma-Aldrich, USA) (CAS-No. 298-93-1). Briefly, cells were seeded at 5 × 10^3^ cells/well in 96-well plates at 37 °C and cultured overnight before treatment with varying concentrations of Amy (0.5–160 mg/ml) and Sor (0.125-8 µM) for 48 h. MTT working solution (100 µl) was added to each well and the plates were incubated in dark at 37 °C for 4 h. The medium was removed and the purple formazan crystals were dissolved by adding 100 µL/well of DMSO for 5 min. The optical density (OD) was measured at an absorbance value of 570 nm using a microplate reader (BIORAD PR4100, USA). Cell viability was calculated in treated cells compared to control untreated cells, which were considered 100% viability and were presented in graphs for the calculation of the 50% inhibitory concentration (IC_50_) [41].

### Combination index (CI) assay

Using MTT assay data, the combination index (CI) was calculated by the Chou-Talalay method as described by Chou et al. using *CompuSyn* software (CompuSyn, Inc., Paramus, NJ, USA) [42]. The dose-effect curves for single and cotreatment were generated and the CI for every dose and the corresponding effect, fraction affected (Fa) were calculated. The resultant CI values reflect the potential interactions between two drugs. CI < 1 indicates synergism, CI = 1 indicates an additive effect and CI > 1 indicates antagonism. The Dose-reduction index (**DRI**) was calculated from the DRI equation and algorithm using *CompuSyn* software, (DRI_50_) values represent the magnitude of dose reduction obtained for the 50% growth inhibitory effect in Amy/Sor cotreatment compared to each drug alone that causes the same growth inhibition effect.

### Evaluation of the antioxidant capacity for Amy and antioxidant markers

The 2, 2-diphenyl-1-picrylhydrazyl (DPPH) (CAS No. 1898-66-4) free radical scavenging method was used to measure the free radical scavenging abilities of Amy as described in a previously published study [43]. The DPPH scavenging activity was calculated using the formula:

$$\mathbf{D}\mathbf{P}\mathbf{P}\mathbf{H}\,\mathbf{S}\mathbf{c}\mathbf{a}\mathbf{v}\mathbf{e}\mathbf{n}\mathbf{g}\mathbf{e}\mathbf{d}\left(\varvec{\%}\right)= \left({\varvec{A}\varvec{B}}_{\varvec{b}\varvec{l}\varvec{a}\varvec{n}\varvec{k}}-{\varvec{A}\varvec{B}}_{\varvec{t}\varvec{e}\varvec{s}\varvec{t}}\right)/{\varvec{A}\varvec{B}}_{\varvec{b}\varvec{l}\varvec{a}\varvec{n}\varvec{k}}\times 100$$

The concentration of the sample leading to a 50% reduction of the initial DPPH concentration (EC_50_) was calculated for Amy and vitamin C from Sigma–Aldrich, USA (CAS No. 50-81-7) as a reference antioxidant.

Assessment of reduced glutathione (GSH) levels in both HepG2 & WI-38 cells lysate treated with half IC_50_ concentrations of Amy and/or Sor for 48 h was performed as described [44].

The concentration of GSH was calculated using a standard curve by the following equation:

GSH concentration (µmol/mg protein) = absorbance / (slope × protein concentration).

Similarly, the Malondialdehyde (MDA) level as a marker of lipid peroxidation was estimated in both HepG2 & WI-38 cell lysate as described in [45].

The MDA concentration was calculated as:

MDA (nM/ml) = (Absorbance/ molar absorptivity) X (1000/sample volume in µl.

### Annexin-V assay

The cell death mechanism was evaluated by flow cytometry using the annexin-V & propidium iodide (PI) double staining apoptosis detection kit (Southern Biotech, Birmingham, AL, USA) following the manufacturer’s instructions. Cells were adjusted to 1 × 10^6^ cells/ml and plated in 6-well plates and allowed to grow for 24 h. Cells were then treated with IC_50/2_ concentrations of Amy, Sor and in combo for 48 h before trypsinization, washing with PBS, and fixation with ethanol for 12 h. Cells were washed with ice-cold culture medium before staining with annexin V-FITC/PI solution and incubation on ice for 10 min. Finally, cells were analyzed using the Accuri C6 flow cytometer (Becton Dickinson, Sunnyvale, CA, USA) [46].

### Cell cycle analysis

For the cell cycle phase distribution analysis assay, HepG2 and WI-38 cells were starved for 24 h before treatment with IC_50/2_ concentrations of Amy, Sor and their combination for 48 h. Treated cells were washed with PBS (Sigma - P2667) and fixed in the dark with 70% pre-cooled ethanol (Sigma –Aldrich, USA, CAS-No. 64-17-5) for 1 h before washing with PBS and subsequent treatment with RNase I at 37 °C for 30 min. Finally, cells were stained with propidium iodide (PI) (Sigma - P2667) at 4 °C for an additional 30 min and analyzed by an Accuri C6 flow cytometer [47].

### RNA extraction and RT-PCR assessment

Total RNA was extracted from half of the IC_50_ concentration treated cells using the Thermo Fisher Scientific, Waltham, MA, USA, Gene JET RNA extraction kit (Cat#K0731), according to the manufacturer’s instructions. The purity and concentration of the extracted RNA were determined by Nanodrop Spectrophotometer (AnalytikJena Scandrop200, Germany) and cDNA was synthesized using SensiFAST™ cDNA Synthesis Kit, Thermo Co, BIO-6505, USA, according to the manufacturer’s protocol.

Quantitative RT-PCR was carried out utilizing specific primers for HMGB1, AMPK, mTOR, BCL2, ATG5, Beclin 1, and LC3 genes, primers sequences are listed in (Supplementary Table 1). The PCR reaction mixture consisted of 10µL SYBR green mix (SensiFAST SYBR No-ROX Mix), 2µL cDNA template, and 6.4 µL nuclease-free water. A rotor gene Q5plex detection system was used for amplification. The thermal cycling condition was as follows: an initial activation for 2 min at 95 °C, followed by 45 cycles of 95 °C for 5s and 62 °C for 10s followed by 72 °C for the 20s. The relative expression was calculated using the comparative 2^−ΔΔCt^ method, GAPDH as an internal housekeeping gene [48].

### *In silico* molecular docking analysis

Three-dimensional structure of human RAC(Rho family)-alpha serine/threonine-protein kinase (AKT1; (PDB ID: 6S9W), AMPK (PDB ID: 4CFF), DNA (cytosine-5)-methyltransferase 1 (DNMT1; (PDB ID: 4WXX), histone deacetylase 1 (HDAC1; (PDB ID: 4BKX), Jumonji domain containing 1 C (JMJD1C; (PDB ID: 2YPD), liver kinase B1 (LKB1; (PDB ID: 4ZDR), phosphatidylinositol-4,5-bisphosphate 3-kinase, catalytic subunit alpha (PK3CA; (PDB ID: 2RD0), and sirtuin 1 (SIRT1; (PDB ID: 5BTR) were retrieved from RCSB-PDB (https://www.rcsb.org/) database. All proteins were prepared for molecular docking by Molecular Operating Environment software (MOE, Chemical Computing Group). Amy and Sor’s three-dimensional structure were downloaded from the PubChem database (https://pubchem.ncbi.nlm.nih.gov/). Molecular docking between receptors and ligands has been performed using MOE software. The interactions between ligands and receptors were visualized using MOE software. Furthermore, ligand efficiency (kcal/mol), dissociation constant (pKd), and inhibition constant (pKi) were calculated by K_DEEP_ (https://playmolecule.com/Kdeep/). Pharmacokinetics, including (ADME-Tox) absorption, distribution, metabolism, and excretion - toxicity of Amy and Sor were determined by ADME and Ames prediction that built in BIOVIA Discovery Studio 2016 (BIOVIA, Dassault Systèmes, France).

### Statistical analysis

Data were analyzed using GraphPad Prism version 8.0 (GraphPad Software, Inc., La Jolla, CA, USA). Data were represented as mean ± SEM, and statistical comparisons between multiple groups were performed in oneway (ANOVA), followed by a Tukey’s post hoc test. For all tests, differences between means were determined by the least significance difference test with significance defined at P ≤ 0.05.

## Results

### Cytotoxicity, drug interaction, and selectivity of Amy and Sor

**Fig. 1:**
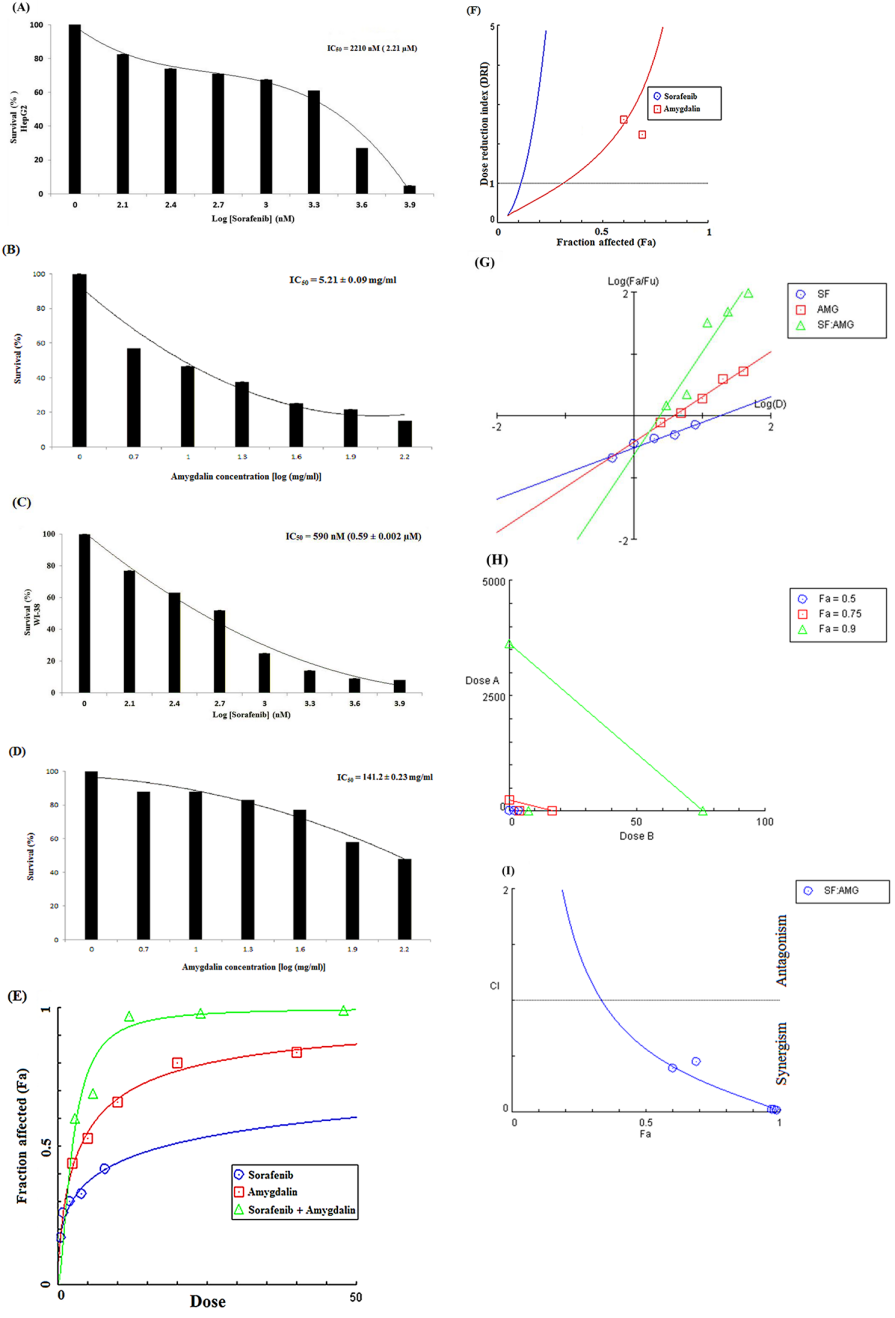
Effect of Sorafenib and Amygdalin on cell viability of *HepG2* and *WI-38* cells: Cells were treated with different concentrations of Sorafenib (0.125-8 µM) **(A and C)** and Amygdalin (0.5–160 mg/ml) **(B and D)** for 48 h and MTT assay was performed. Results were expressed as cell viability (% of control) and data are presented as the mean ± standard deviation, all experiments were repeated at least three times. **(E-I)** Graphic representations obtained from the *CompuSyn* Report (see supplementary materials 2) for Amy and Sor combinations indicating strong synergism between the two compounds when used in combo: **(E)** Dose-effect plot; **(F)** DRI plot; **(G)** Median-effect plot; **(H)** Isobolograms; and **(I)** Combination index blot

The cytotoxic potential of Amy and Sor was evaluated against HepG2 and WI-38 cells using an MTT test. 48 h were spent treating cells with Amy and Sor (range: Amy: 0.5–160 mg/ml; Sor: 0.125-8 µM). Both substances showed dose-dependent reductions in cell viability in both cell lines. Amy inhibited the growth of HepG2 (IC_50_ 5.21 ± 0.09 mg/ml) and WI-38 (IC_50_ 141.25 ± 0.23 mg/ml). Sor reduced the proliferation of WI-38 cells (IC_50_ 0.59 ± 0.002 µM) and HepG2 cells (IC_50_ 2.21 ± 0.06 µM) like that of HepG2 Fig. 1.

On HepG2 cells, the potential for pharmacological interactions (synergistic, additive, or antagonistic) between combinations of Amy and Sor were evaluated by MTT assay and analyzed by *CompuSyn* software (see supplementary materials 2 for complete report). The *CompuSyn* blots are illustrated in Fig. 1**(E-I)**. Cells co-treated with different doses of Amy and Sor showed significantly decreased viability than the comparable single therapy. Additionally, the DRI_50_ for Amy and Sor were equivalent to 1.8 and 43.5, respectively (Table 1). When the two compounds were administered in combination at 2.036 mg/ml Amy and 0.40 µM Sor for 48 h, they resulted in a 0.5 fraction inhibition (Fa) and a strong synergistic interaction (CI_50_ = 0.56), the CI values ranged from 0.566 to 0.8 for the fractional inhibition of Fa = 0.50 ~ 0.97 (Table 2) (Supplementary File 1). The synergism was further substantiated by the isobolograms (Fig. 1H), and the Fa-DRI (Fig. 1F), and Fa-CI plots (Fig. 1I), which evaluated the possibility of dosage reduction and the impact of Amy and Sor’s co-treatment, respectively.

**Table 1:** Combination index (CI) and dose reduction index (DRI) values for Amy and Sor combinations in HepG2 cells

Cell line	Dose	DRI_50_	CI_50_	Interpretation
HepG2	Amygdalin: 2.04 mg/ml	1.8	0.56	Strong Synergism
	Sorafenib: 0.74 µM	43.5		

CI_50_ is the combination index for the 50% effect

**Table 2:** Synergistic effect of Amygdalin and Sorafenib against HepG2 cell growth after 48 h treatment

	Fraction Affected (Fa)	Dose		CI ^a^	DRI ^b^	
		**Amy** **(mg/ml)**	**Sor** **(µM)**		**Amy**	**Sor**
Combination	0.5	2.03629	0.40726	0.56644	1.83994	43.5887
	0.75	3.95499	0.79100	0.23784	4.26039	320.631
	0.9	7.68158	1.53632	0.10179	9.86495	2358.51
	0.95	12.0653	2.41307	0.05737	17.4625	9163.72
	0.97	16.6365	3.32730	0.03819	26.2164	24069.5

Dose and effect data were obtained from the MTT assay and were analysed by *CompuSyn* software. This Table was created using data produced by *CompuSyn* Report. ^**a**^ Combination index **(CI)** was calculated from the CI equation algorithms using *CompuSyn* software. CI = 1, < 1 and > 1 indicates additive, synergistic and antagonistic effect, respectively. ^**b**^ Dose-reduction index **(DRI)** was calculated from the DRI equation and algorithm using CompuSyn software. DRI = 1, > 1, and < 1 indicates no, favourable, and not favourable dose-reduction, respectively, for every drug in the corresponding combination

The Selectivity Index (SI) was calculated using the ratio of the IC50 values for Amy and Sor versus HepG2 and the normal WI-38 cells (Table 3). Amy was particularly toxic for HepG2 cells (SI = 27.1), but not toxic for WI-38 normal cells. However, both cell lines were severely damaged by Sor, especially the WI-38 cells (SI = 0.27).

**Table 3:** Calculated IC_50_ and selectivity index values for Amy and Sor against HepG2 and WI-38 cell lines

Treatment	IC_50_		Selectivity Index (SI)
	HepG2	WI-38	
**Amygdalin (**mg/ml)	5.21 ± 0.09	141.25 ± 0.23	27.1
**Sorafenib (**µM)	2.21 ± 0.06	0.59 ± 0.002	0.27

### Effect of Amy and Sor on the morphology of HepG2 and WI-38 cells

**Fig. 2:**
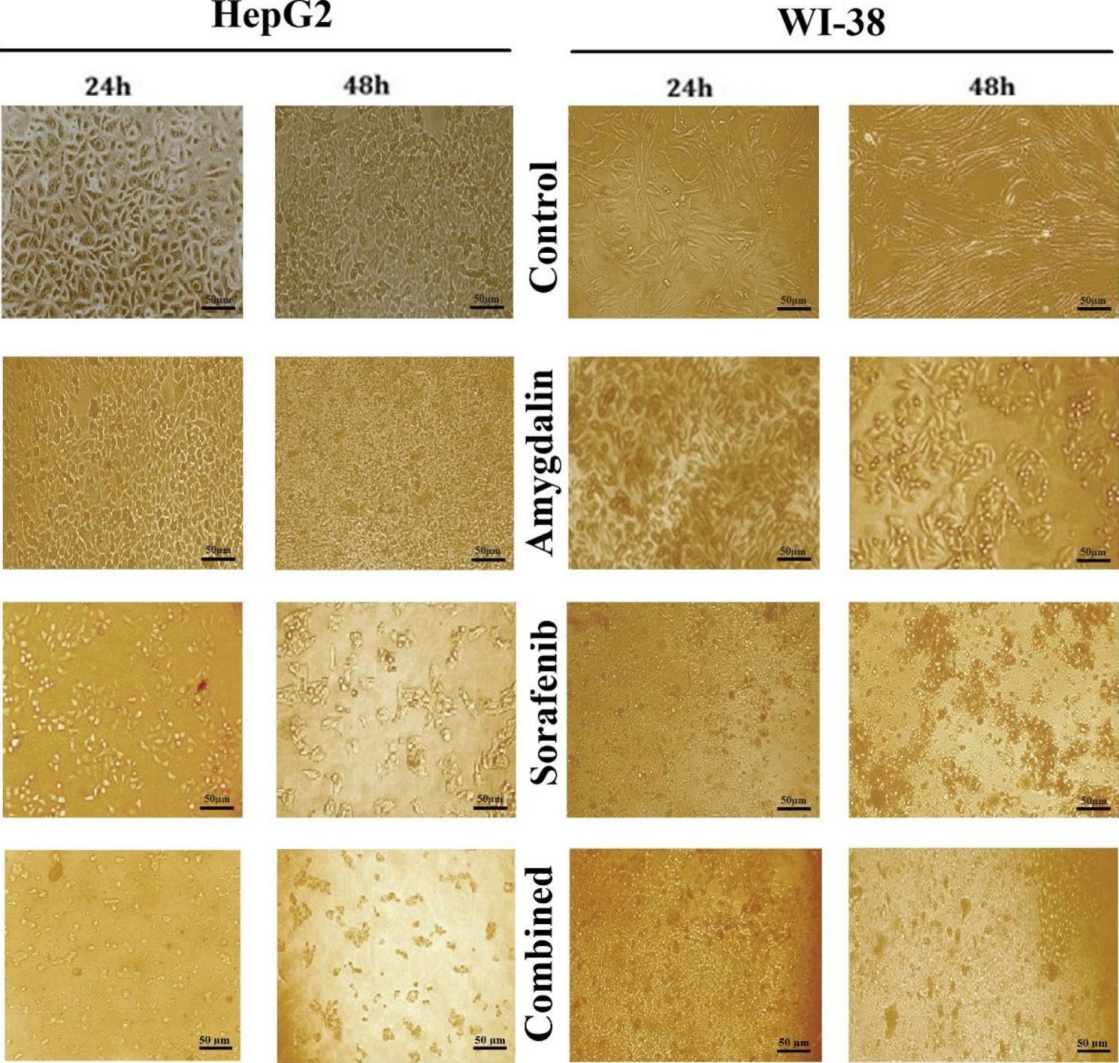
Representative photographs showing morphological changes in HepG2 and WI-38 cells exposed to IC_50/2_ concentration of Amygdalin or Sorafenib and combination treatment for 24 and 48 h. Images were taken using an inverted phase contrast microscope at ×100. Scale bar 50 μm

Under the inverted phase-contrast microscope, changes in HepG2 and WI-38 cell morphology were seen after being exposed to the IC50 concentrations of Amy or Sor for 24 and 48 h, respectively Fig. 2. Untreated control cells showed normal morphology, however, cells that had been exposed to Amy and/or Sor showed abnormal morphology and characteristic signs of cell death, such as fragmented nuclei, rounded membrane deformations, decreased cell density, and the emergence of clusters of floating dead cells.

### Amy induces apoptosis and ameliorates the Sor-induced necrosis in HepG2 cells

**Fig. 3:**
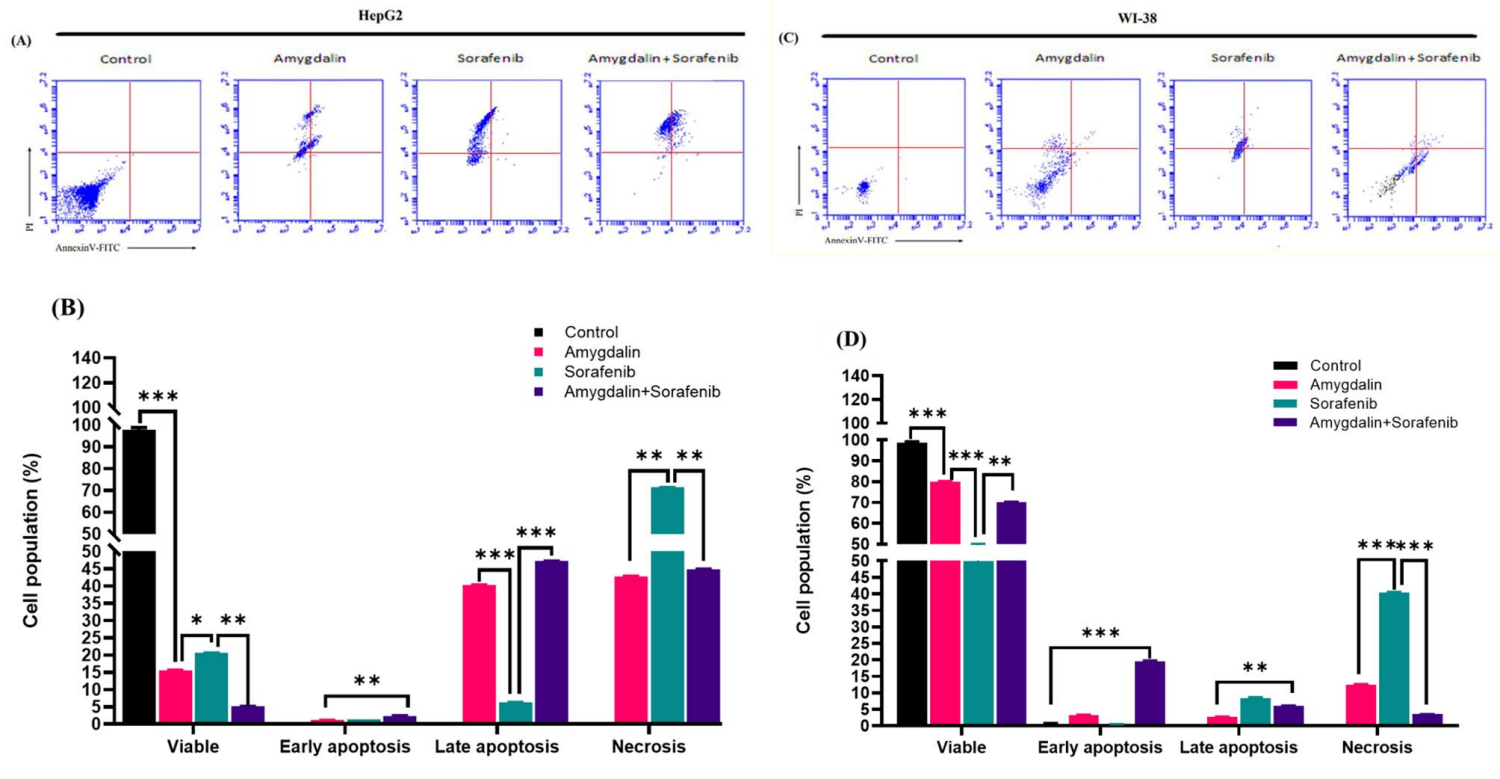
Representative dot plots of cell apoptosis: **(A)** HepG2 and **(C)** WI-38 cells treated with IC_50/2_ concentration of amygdalin or sorafenib alone and in combination for 48 h before double staining with annexin V-FITC/PI and analysis by flowcytometry. The percentage of live cells (AnnexinV-FITC− /PI −), early apoptotic (AnnexinV-FITC+ /PI −), late apoptotic (AnnexinV-FITC+ /PI +), and necrotic cells (AnnexinV-FITC− /PI +) were measured, represented graphically in **(B)** and **(D)**. Data are represented as mean ± SEM (n = 3; *significantly different as compared to the untreated control group; *** (P ≤ 0.001), ** (P ≤ 0.01),* (P ≤ 0.005)

The loss in cell viability could be brought about by an apoptotic response to Amy and Sor, as suggested by the MTT assay and microscopic inspection. To further establish the cell death process (apoptosis vs. necrosis), both HepG2 and WI-38 cells were treated with half IC50 doses of each chemical for 48 h. Figure 3.

In untreated control, HepG2 cells, the percentages of viable, early, late, and necrotic cells were 99.8%, 0.2%, 0.0%, and 0.0%, respectively. The proportion of late apoptotic cells, however, significantly increased following 48 h of treatment with Amy, Sor and in combination, reaching 40.1%, 6.5%, and 47.4%, respectively. Similar to this, the proportion of necrotic cells was 43.0% in Amy-treated cells, 71.8% in Sor-treated cells, and 45.2% in Amy/Sor-cotreated cells. According to the cytotoxic synergistic impact, only 5.4% of viable cells were found in the Amy/Sor combination therapy, compared to 15.7% in the Amy and 20.3% in the Sor single treatment .

Compared to HepG2 cells, WI-38 normal cells responded to treatment somewhat differently. In general, WI-38 cells revealed more live and early apoptotic cells than HepG2 cells did when given the same therapy. When compared to the control (99.3%), a single treatment with Sor generated the lowest percentage of viable cells (50.2%) and the highest late apoptotic (9.0%) and necrotic cells (40.2%). Our findings show that Amy and Sor work together to kill cancer cells by triggering late apoptosis in HepG2 cells (Supplementary Fig. 1).

### Effect of Amy and Sor on the cell cycle distribution

**Fig. 4:**
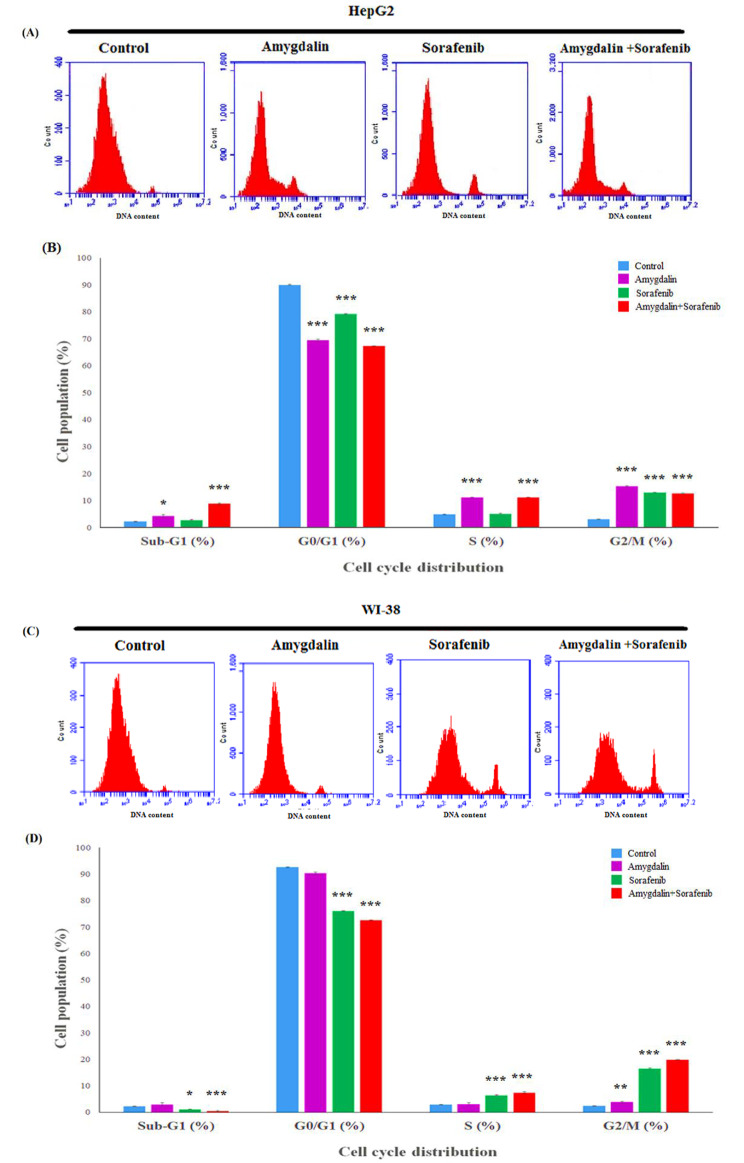
Effect of amygdalin, sorafenib, and their combination on cell cycle. Flow cytometry analysis of **(A)** HepG2; **(C)** WI-38 cells after treatment with IC_50/2_ concentration for each drug for 48 h. (**B, D**) Quantitative data analysis for the cell population (% of total) in Sub-G1 G_0_/G_1_, S, and G_2_/M phases. Results are presented as mean ± SEM (n = 3; *significantly different as compared to the untreated control group; *** (P ≤ 0.001), ** (P ≤ 0.01), and * (P ≤ 0.05)

Using flow cytometry, the distribution of the cell cycle phase was examined in Fig. 4. After treatment with Amy (11.1 ± 0.11%) and combined Amy/Sor (11.2% ±0.11), HepG2 cells showed a considerably high accumulation of cells, indicating a cell cycle arrest at the S phase after treatment with Amy (11.1 ± 0.11%) and combined Amy/Sor (11.2%±0.11) as compared to control (4.8%±0.11). Amy’s treatment of HepG2 cells resulted in a notable five-fold rise in the population of G2/M cells (15.3% ±0.05) as compared to the control (3% ±0.3). After combining Amy/Sor and Sor’s single treatments, the normal WI-38 cell line showed the greatest levels of cell cycle arrest in the G2/M and S phases (Supplementary Fig. 2).

### Effect of Amy and Sor on signaling pathways and apoptosis-autophagy-related marker genes

**Fig. 5:**
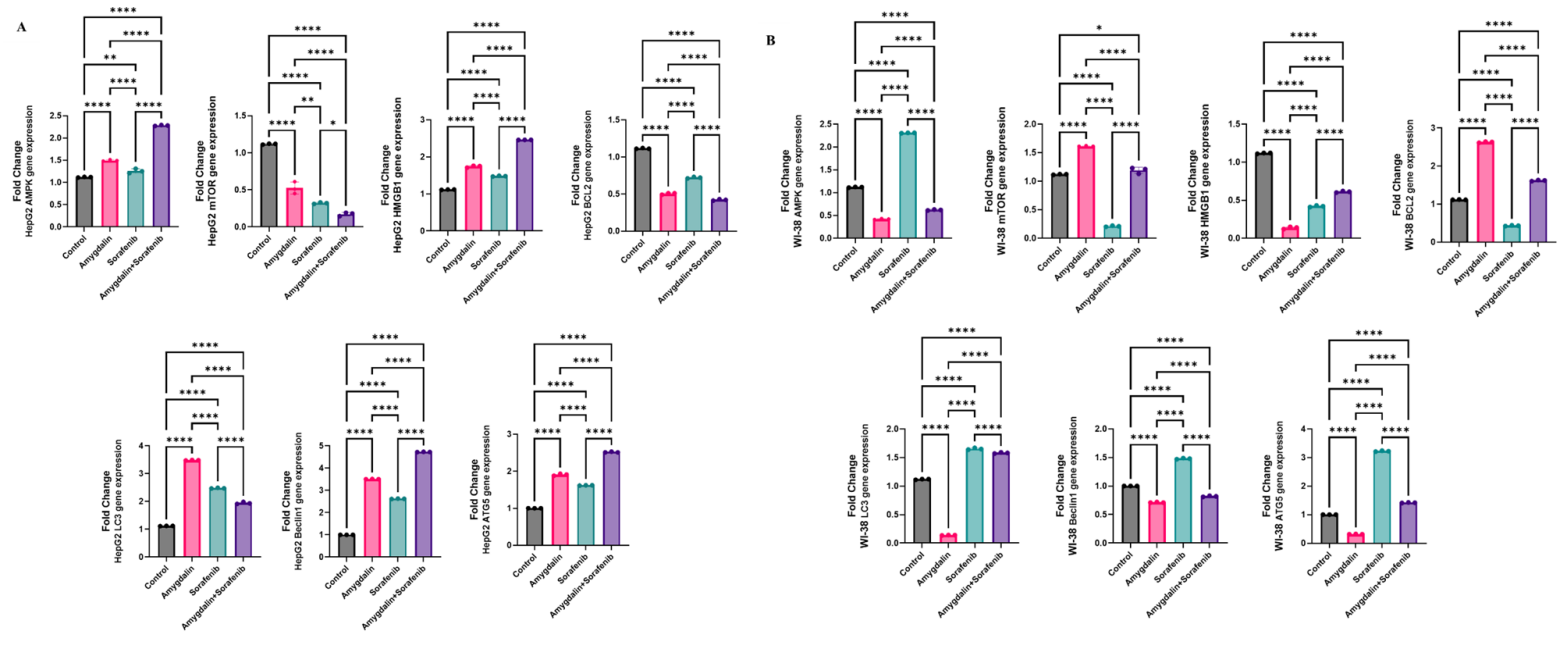
Effect of Amygdalin or Sorafenib single and combined treatment on expression of apoptosis-autophagy related genes: **(A)** HepG2; **(B)** WI-38 cells. Results are presented as mean ± SEM (n = 3). * (P ≤ 0.05), ** (P ≤ 0.01) *** (P ≤ 0.001) compared to control

RT-PCR was used to analyze the impact of Amy and Sor on the expression of the genes for AMPK and mTOR as well as many apoptosis-autophagy-related indicators, including HMGB1, BCL2, LC3, Beclin 1, and ATG5. In HepG2 cells, as depicted in Fig. 5A, combined Amy/Sor treatment caused the most prominent upregulation of AMPK, HMGB1, Beclin 1, and ATG5 expression, whereas mTOR and BCL2 expressions were most strongly suppressed when compared to control and single Amy or Sor treated HepG2 cells. As opposed to control, single Amy, or combination Amy/Sor therapy, Sor single treatment substantially (p < 0.05) caused the highest expression of AMPK, LC3, Beclin 1, and ATG5, along with the highest suppression for mTOR and BCL2 genes in WI-38 cells Fig. 5B.

### Free radical scavenging capacity and oxidative stress markers

**Fig. 6:**
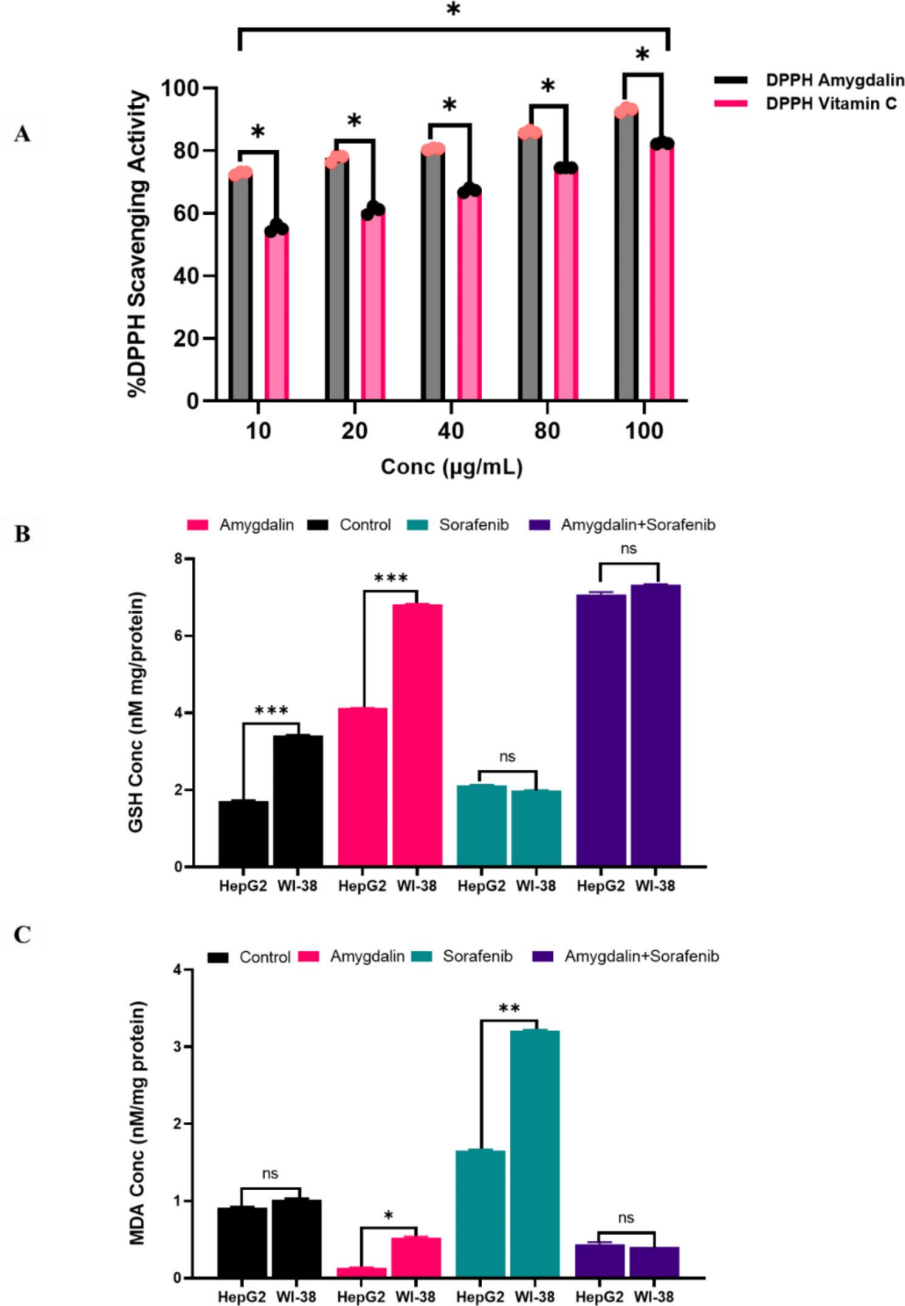
Scavenging activity of AMG and effect of AMG and SOR single and combined treatments on oxidative stress markers: **(A)** DPPH scavenging activity of AMG versus the standard vitamin C; **(B)** GSH and **(C)** MDA concentration in control and treated HepG2 and WI-38 cells. * (P ≤ 0.05), ** (P ≤ 0.01) and *** (P ≤ 0.001) means significantly different compared to control. All analyses were performed in triplicate and data are expressed as mean ± SD.

The reaction of the dry DPPH dissolved in methanol to various doses of vitamin C and Amy (5–75 mg/ml and 2.5–25 µg/ml, respectively) is depicted in Fig. 6A; the amount of DPPH that Amy was able to scavenge increased dose-dependently. Amy and vitamin C each had an EC50 of 15.64 mg/ml and 178.5 g/ml, respectively. As such, treated cells had much higher GSH levels than the untreated control cells. HepG2 and WI-38 cells with Amy/Sor combination therapy had the greatest GSH levels, as demonstrated in Fig. 6B. However, in HepG2 and WI-38 cells, the combination of Amy and Sor therapy resulted in the most significant reduction in MDA levels when compared to the control. In contrast, HepG2 (1.89 nM/mg ± 0.05) and WI-38 (3.12 nM/mg ± 0.1) cells treated with Sor alone had significantly higher MDA levels than control cells Fig. 6C.

### Molecular docking analyses predict the cellular target proteins of Amy and Sor

**Fig. 7:**
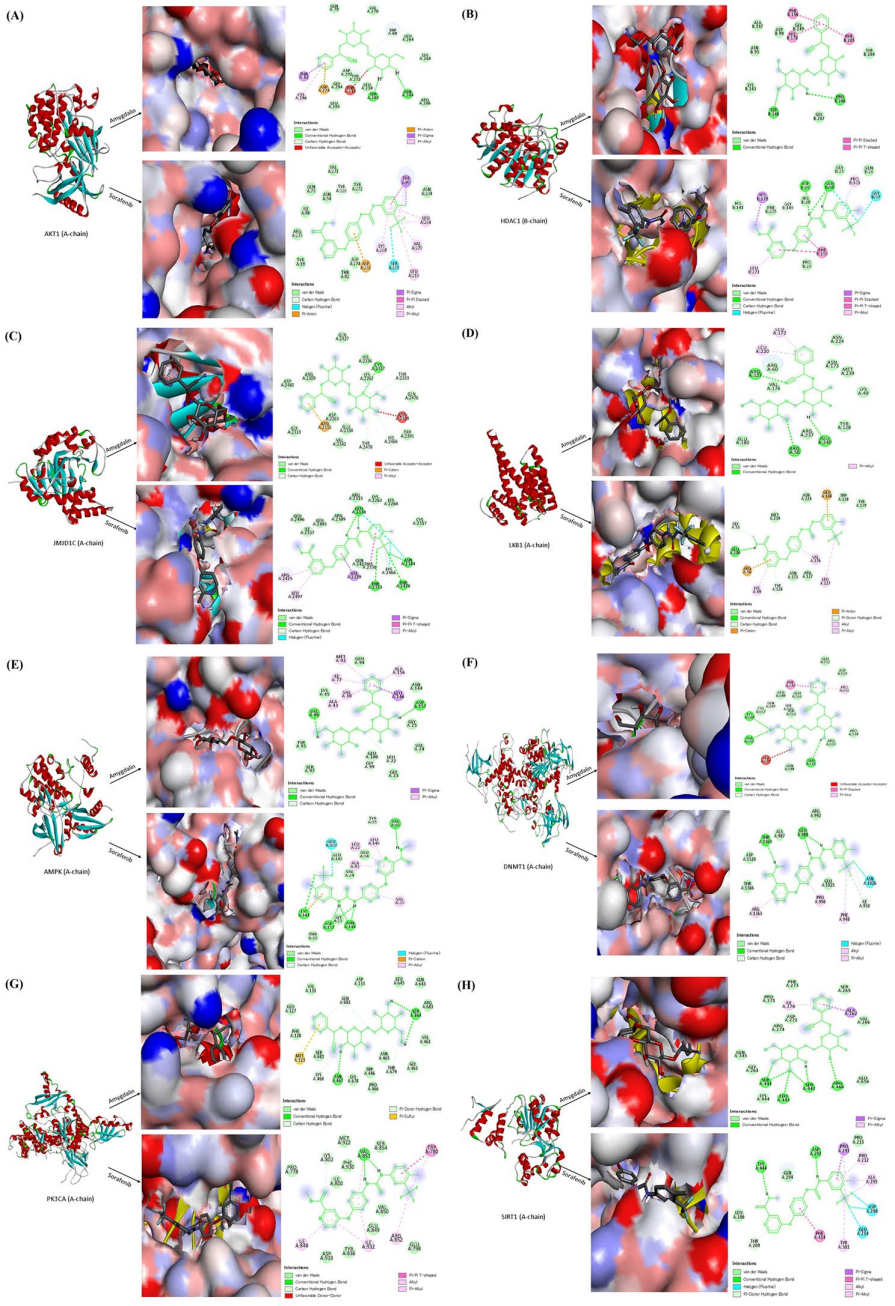
Molecular docking analysis for the visualization of binding poses of amygdalin and sorafenib in the binding sites of **(A)** AKT1 (PDB ID: 6S9W); **(B)** HDAC1 (PDB ID: 4BKX); **(C)** JMJD1C (PDB ID: 2YPD); **(D)** LKB1 (PDB ID: 4ZDR); **(E)** AMPK (PDB ID: 4CFF); **(F)** DNMT1 (PDB ID: 4WXX); **(G)** PK3CA (PDB ID: 2RD0) and **(H)** SIRT1 (PDB ID: 5BTR). Molecular docking and interactions between ligands and receptors have been performed using InstaDock and BIOVIA discovery studio visualizer software, respectively. Amygdalin and sorafenib three-dimensional structure were downloaded from the PubChem database (https://pubchem.ncbi.nlm.nih.gov/)

Amy and Sor were screened for probable targets using the Swiss target prediction algorithm. These proteins have been identified as having a high binding probability, and virtual screening was used to further narrow them down to those with the highest affinity and most promise for modulating the processes associated with cancer progression. The results of the screening suggest that these eight proteins [AKT1, AMPK1, DMNT1, HDAC1, JMJDIC, LKB1, PK3CA, and SIRT1] may have the potential to effectively target cancer progression. The measurement of a compound’s free binding energy (measured in Kcal/mol) allows molecular docking to forecast the compounds that have the highest chance of forming a strong bond with a protein. Each receptor was allowed to dock with several ligand poses, which were then analyzed based on the binding energy of each docking position. Amy has been molecularly docked to PI3K and mTOR proteins to examine its potential impact as an AMPK/PI3K/mTOR inhibitor. Our findings in this investigation suggested that Amy/Sor had greater binding energies for AKT1 (-7.12 kcal/mol and − 7.05 kcal/mol, respectively), which made them good candidates for the antagonistic treatment of angiogenesis and cell proliferation. Interestingly, the interaction between Amy and the ATP binding site AKT resembles the Sor ligand-protein complex in certain ways Fig. 7A. Furthermore, Amy also showed binding energies with a variety of receptors, including HDAC1 (-5.63 kcal/mol), JMJD1C (-6.8 kcal/mol), and LKB1 (-5.79 kcal/mol). However, Amy and Sor have demonstrated strong binding energy with AMPK (-5.75 and − 6.43 kcal/mol), DNMT1 (-5.89 and − 6.50 kcal/mol), PK3CA (-6.33 and − 7.03 kcal/mol), and SIRT1 (-6.66 and − 6.35 kcal/mol), respectively (Fig. 7B-H). Furthermore, the dissociation constant (pKd) and inhibition constant (pKi) of Amy and Sor toward the target proteins are represented in (Table 4). Amy and Sor were determined by ADME and Ames prediction and the results were tabulated in (Table 5).

**Table 4:** Molecular docking scores of Amy and Sor against human RAC(Rho family)-alpha serine/threonine-protein kinase (AKT1; PDB ID: 6S9W), AMP-activated protein kinase (AMPK; PDB ID: 4CFF), DNA (cytosine-5)-methyltransferase 1 (DNMT1; PDB ID: 4WXX), histone deacetylase 1 (HDAC1; PDB ID: 4BKX), jumonji domain containing 1 C (JMJD1C; PDB ID: 2YPD), liver kinase B1 (LKB1; PDB ID: 4ZDR), phosphatidylinositol-4,5-bisphosphate 3-kinase, catalytic subunit alpha (PK3CA; PDB ID: 2RD0), and sirtuin 1 (SIRT1; PDB ID: 5BTR)

	Amygdalin			Sorafenib		
	S score^¥^ (kcal/mol)	pK*d*^§^	pK*i*^§^	S score^¥^ (kcal/mol)	pK*d*^§^	pK*i*^§^
**AKT1**	-7.12	5.64 ± 0.58	5.78 ± 0.67	-7.05	6.58 ± 0.34	6.59 ± 0.60
**AMPK**	-5.75	5.09 ± 0.30	5.48 ± 0.27	-6.43	6.51 ± 0.40	6.64 ± 0.45
**DNMT1**	-5.98	4.47 ± 0.45	4.62 ± 0.46	-6.50	5.93 ± 0.43	6.15 ± 0.33
**HDAC1**	-5.63	4.01 ± 0.34	4.15 ± 0.41	-5.08	5.03 ± 0.37	5.38 ± 0.42
**JMJD1C**	-6.80	5.62 ± 0.50	6.00 ± 0.56	-6.46	6.38 ± 0.71	6.63 ± 0.54
**LKB1**	-5.79	4.64 ± 0.53	5.22 ± 0.80	-5.50	5.20 ± 0.52	5.07 ± 0.65
**PK3CA**	-6.33	5.10 ± 0.52	5.07 ± 0.65	-7.03	6.70 ± 0.46	7.14 ± 0.38
**SIRT1**	-6.66	5.71 ± 0.65	5.81 ± 0.74	-6.35	5.52 ± 0.22	6.01 ± 0.58

pK*d*; dissociation constant. pK*i*; inhibition constant. Mean ± SD.

^¥^docking score was determined by Molecular Operating Environment software (MOE) software

^**§**^pK*d* and pK*i* were determined by K_DEEP_ server

**Table 5:** Absorption, distribution, metabolism, and excretion (ADMET) and Ames’s prediction scores of amygdalin and sorafenib

	ADMET										TOPKAT Ames			
	Solubility	Solubility level	Absorption level	BBB level	CYP2D6	Hepatotoxicity	Hepatotoxicity prediction	PPB	PPB prediction	AlogP98	Prediction	Probability	Enrichment	Scores
Amygdalin	-0.924	4	3	4	-4.92333	-15.707	FALSE	-15.6493	FALSE	-2.403	Non-Mutagen	0.134463	0.240814	-16.0437
Sorafenib	-6.059	1	0	4	-6.28535	-0.741939	TRUE	6.00289	TRUE	4.175	Non-Mutagen	0.0531248	0.0951423	-19.6956

TOPKAT; toxicity prediction. ADMET; absorption, distribution, metabolism, and excretion

## Discussion

The development of novel chemotherapeutic medicines is crucial in the fight against liver cancer [49], which is regarded as the third greatest cause of mortality worldwide [50]. For patients with advanced-stage HCC, Sor has been the most important molecular targeted medicine [51], but it hasn’t responded to either curative interventional or chemotherapies [52], can’t extend overall survival past three months, and over 50% of patients experience severe clinical side effects [53]. Additionally, Sor medication resistance significantly diminishes the treatment’s efficacy in patients [54]; Sor dose attenuation is required due to all of these considerations [55]. As a result, phytoconstituents that exhibit antioxidant, anti-cancerous, and hepatoprotective properties are of significant interest to oncologists [56]. The ADMET results of the current study also, stated the toxicity of Sor despite its anticancer potentials. Therefore, the idea behind this combination is to reduce the toxic effects of Sor by introducing a natural product such as Amy that may be better tolerated by the body. This could potentially increase the effectiveness of the combination while reducing the potential side effects. Several in vitro and in vivo studies on Amy have been conducted to gain insight into its biological function and potential therapeutic targets for HCC [57, 58]. In this work, Amy and Sor’s antiproliferative activities against HepG2 cancer cells and the non-cancerous WI-38 cell line were assessed both singly and in combination to determine whether Amy is potentially cancer-selective and whether it is safe for normal cells; a review of the tested medications’ ability to cause autophagy-induced programmed cell death, which has not previously been properly evaluated.

The MTT assay is a useful test that accurately determines the number of viable cells and may be used to assess the cytotoxicity of substances used in the treatment of cancer [59]. The MTT results show that Amy decreases the HepG2 cell line’s viability in a dose-dependent manner. Similar to our study, other researchers have also documented the cytotoxicity effect of Amy in a variety of cancer cells, including oral cancer cell line [60], breast cancer [61], and human cervical cancer [62]. This effect could be explained by the morphological changes in HepG2 cells that lose their epithelial shape, which makes them appear smaller, spherical, with a disfiguration in the cell membrane, and detached from the surface when compared to untreated ones, which is a sign of cell death as described in the literature.

Our result revealed that Sor showed a low selectivity index (SI) value (0.27) versus the 100 folds higher SI value observed for Amy (27.1). As previously clarified in many published literature the higher the (SI) value, the greater drug selectivity as it reflects a higher drug normal IC50 relative to cancer; where (SI) value less than 2; suggested general drug toxicity [63–65]; such results confirmed Sor toxic effect; representing Amy as a good choice therapy with high selectivity for HCC accomplished by no toxic side effects.

Our data showed that our co-treatment had a strong synergistic effect, as indicated by the CI analysis, which had a value equal to (0.65), indicating strong synergism. This synergistic effect was beneficial for reducing the doses of both Sor and Amy, as demonstrated by the DRI_50_ value, which calculates how many folds the dose of each drug in a synergistic combination may be reduced, with DRI_50_ 1 being more favorable. In addition, a significant dosage decrease was noted in Sor, where the DRI_50_ value was (43.5 with a lowered dose from 17.75 µM to 0.74 µM), and the DRI_50_ value for Amy was (1.8 with a reduced dose from 3.74 mg/L to 2.04 mg/L). However, this potency reduction could be the result of Amy’s naturally multi-targeting bioactive characteristics [66]. In this study, the neutralizing effect of Amy/Sor was seen in both MDA and GSH levels, with considerable augmentation of GSH in cells treated with Amy/Sor cotreatment (6.94 & 7.64), followed by single Amy (4.19 & 6.85), and accomplished with Sor (2.28 & 2.03 in HepG2 and WI-38 cells, respectively. However, MDA significantly decreased in cells treated with Amy (0.04 & 0.26), while it increased in cells treated with Sor (1.89 & 3.12) in HepG2 and WI-38 cells, respectively. This indicates that Amy has a better effect on Sor’s toxic effects when combined, which is a promising attribute in overcoming Sor’s toxic effects. These results were consistent with several previous investigations [67, 68].

Cell cycle analysis is a crucial test that demonstrates the proportion of cells that accumulate in each phase during cell proliferation after exposure to any harmful substance [69]. To ascertain if Amy/Sor affects cell cycle progression and/or the activation of apoptosis, Annexin V-FITC/PI labelling was carried out on HepG2 cells treated with Amy/Sor. Our data showed that all groups slowed the progression of the HepG2 cell cycle in the S and G2/M phases. However, single Amy and/or Sor is the most stressful effect among the groups, inducing the death of HepG2 cells with a drastic increase in the population of Sub-G cells. Besides, the greatest incidence of S and G2/M arrest was seen by Amy or Amy/Sor with the least stressful effect on normal cells. However, various research indicates that Amy and Sor alone were able to halt the cell cycle at various phases after treating distinct cell types [70, 71].

According to our findings, Amy and/or Sor caused HepG2 cells to undergo both necrosis and apoptosis. Cotreatment markedly increased apoptosis, followed by Amy and Sor. Additionally, Sor had the highest rate of necrotic cell death, whereas Amy had the lowest rate. This finding might be explained by MTT and CI data, which show that the combination of Amy and/or Sor played crucial roles in the reduction of HepG2 cell proliferation via both apoptosis and necrosis pathways with the least toxic impact in comparison to a single Sor treatment. The antiproliferative and docking mechanism of Amy further supports these findings.

The ability of cancer cells to avoid apoptosis, or programmed cell death, is one of their well-known traits [72]. Additionally, in cancer cells, autophagy suppresses tumorigenesis by inhibiting cancer-cell survival and inducing cell death. Consequently, further gene analysis for intrinsic and extrinsic genes that regulate apoptosis and autophagy was carried out in our study. The results showed that the Amy/Sor co-therapy led to the upregulation of AMPK, HMGB1, Beclin 1, and ATG5 expression while simultaneously significantly suppressing the expression of mTOR and BCL2. These results may be supported by the fact that mTOR/BCL-2, which is classified as an anti-apoptotic protein because of its function in the production of cell death, controls the apoptotic pathway. Here, we show that Amy causes the HepG2 cell line to go into apoptosis by down regulating BCL-2. Other investigations in numerous cancerous cells have demonstrated that Amy can cause apoptosis via lowering mTOR/BCl-2 [61, 73]. However, sustaining apoptosis depends on the equilibrium between cell division and death [74].

New targeted therapeutics that can either induce death or make cancer cells more susceptible to known cytotoxic drugs have been developed as a result of our growing understanding of the processes of intrinsic and extrinsic apoptotic signaling [75]. Amy causes apoptosis, demonstrating that it blocks the AMPK/mTOR pathway’s activation. The results of this study revealed that the activation of the AMPK signaling pathway was meritoriously inhibited in HepG2 cells and that the translocation of mTOR and BCL2 was significantly suppressed in Amy/Sor-treated cells, suggesting that the AMPK inhibition mechanism may be involved in apoptosis. This behaviour may be explained by the critical function of AMPK as a metabolic sensor and regulation of cell growth [76]; besides, AMPK negatively regulates the mTOR signal pathway, resulting in the inhibition of cancer proliferation and growth [77]; by incorporating signals from the PI3K/Akt pathway [11], it controls cell survival, proliferation, and angiogenesis [78]; additionally, HCC typically has elevated mTOR, which is linked to an early recurrence and a worse prognosis [79]; treatment for HCC has been proposed as the inactivation of mTOR to limit cancer cell growth [80].

**Fig. 8:**
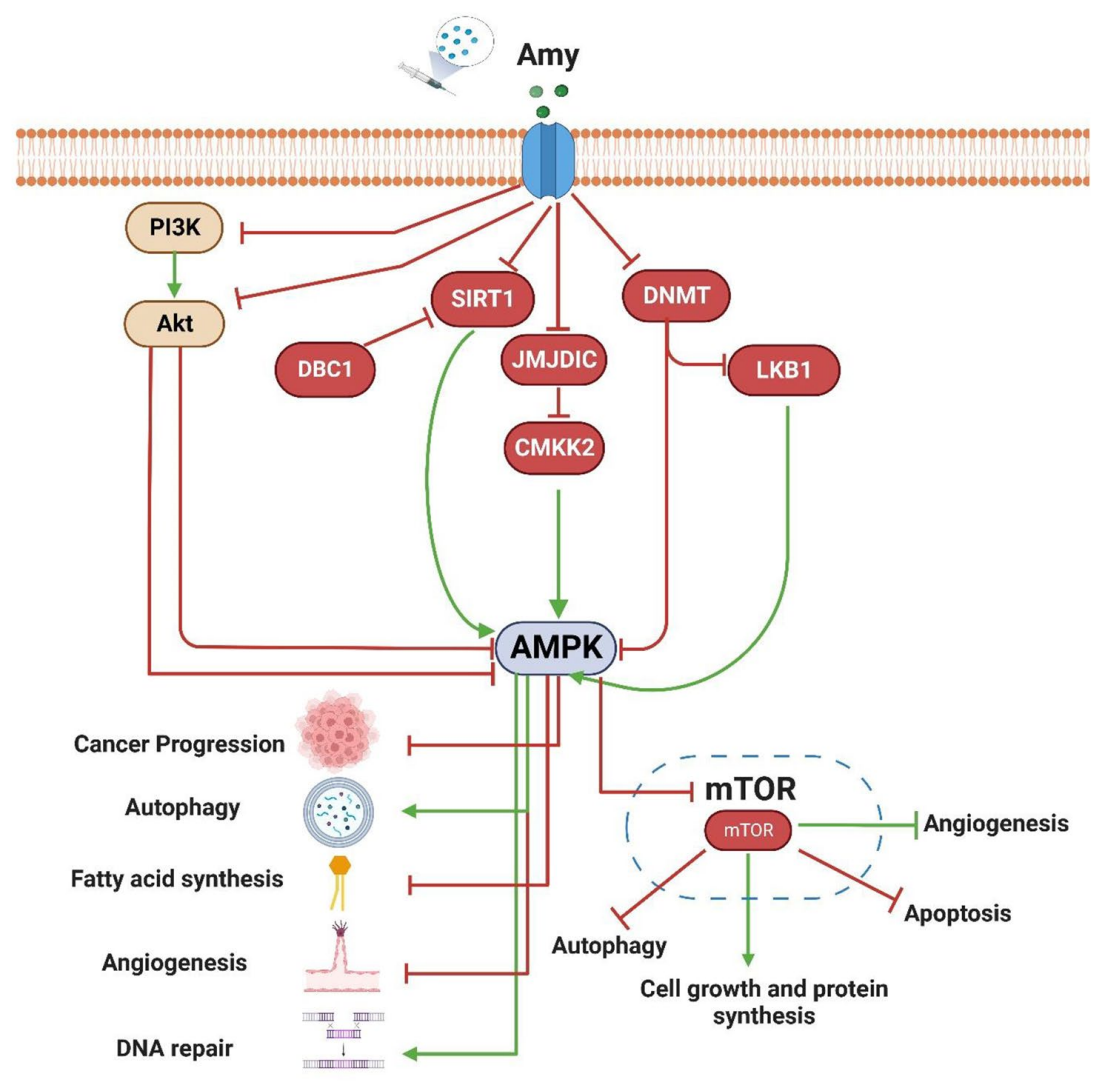
A schematic diagram shows the mode of action of Amy on the HepG2 cell line. Treatment of the HepG2 cell line with Amy resulted in potential cytotoxicity, and significant down-regulation for the AMPK/mTOR/BCL-2 pathway, with induction of apoptosis and autophagy by extrinsic and intrinsic pathways

Consequently, these results are consistent with our theory that Amy induces autophagy through AMPK/mTOR/BCL2 signaling and that autophagy is a critical factor in Amy-mediated cell death [81]. Figure 8 recapitulates the proposed antiproliferative and apoptotic action of Amy on HepG2 cells by up-regulation expression of AMPK, HMGB1, Beclin 1, LC3, and ATG5, and down-regulation expression of mTOR/BCL2 genes that were induced by Amy relative to control and simulate the induction of intrinsic and extrinsic apoptosis pathway in the treated liver cancer cell line.

Interestingly, the proposed antiproliferative and apoptotic action of Amy on HepG2 has been confirmed by molecular docking of anticipated cellular proteins like AKT1, AMPK, DNMT1, HDAC1, JMJD1C, LKB1, PK3CA, and SIRT1 as possible targets of both Amy and Sor. These target proteins play crucial roles in the junction of apoptotic and autophagy crosstalk. Most of the selected proteins in the in-silico study could be inhibited by Amy leading to downregulation of AMPK and subsequently downregulated mTOR. By inhibiting PI3K/AKT, Amy disrupts the pathway that would normally activate mTOR, meaning that mTOR is unable to activate the downstream processes that would lead to cancer progression. In addition, by downregulating AMPK, Amy reduces the amount of AMP in the cell, which in turn reduces the amount of mTOR available to activate the downstream pathways. However, molecular docking proposed the inhibitory action of Amy as follows; the AMPK gene was upregulated as a result of Amy’s inhibition of (a) AKT activity (a repressor for AMPK) by docking the AKT protein and indirectly by docking the PI3K (an enhancer for AKT); (B) Methylation of the AMPK and LKB1 (activator for c) promoter area by docking the DNMT1 enzyme, resulting in direct and indirect increased expression of AMPK. (C) Histone demethylase JMJDIC (the negative regulator for AMPK) by direct docking into the active site. JMJDIC suppresses the CAMKK2 gene, which ordinarily results in the overexpression of AMPK; (D) Binding of DBC1 inhibitor to SIRT1 (AMPK activator), causing sequential activation of SIRT1and LKB1 which in turn causes AMPK activation.

## Conclusion

In this study, we showed that Amy and/or Sor have an anti-proliferative impact and apoptotic action on hepatocellular carcinoma HepG2 cells. Together, the AMPK/mTOR inhibitory signalling pathway contributes significantly to HCC. This study reveals the potential apoptotic anti-HCC properties of Amy and/or Sor via inhibition of AMPK/mTOR. Cytotoxic activity of Amy and/or Sor exhibited that Amy inhibited the growth of HepG2 (IC_50_: 5.21–0.09 mg/ml). Furthermore, a strong synergistic interaction between Amy and Sor (CI_50_ = 0.56) was detected; additionally, the DRI_50_ for Amy and Sor were equal to 1.8 and 43.5, respectively. For investigation of the apoptotic activity, Amy significantly stimulated apoptotic HCC cell death; after 48 h of treatment with Amy and/or Sor, the percentage of late apoptotic cells increased remarkably to 44.9%, 34.8%, and 59%, respectively. However, the greatest levels of mTOR and BCL2 suppression are concurrent with the elevation of AMPK, HMGB1, Beclin 1, LC3, and ATG5 expression in Amy and/or Sor-treated HepG2 cells. In this work, the AMPK/mTOR signalling pathway of Amy was examined using integrated techniques in vitro and *in silico*. Our findings taken together provide more evidence for Amy’s potential anticancer efficacy as a different therapy option for HCC, although additional in vivo studies in animal models are required to corroborate the results.

### Electronic supplementary material

Below is the link to the electronic supplementary material.

Supplementary Material 1

Supplementary Material 2

Supplementary Material 3

Supplementary Material 4

## Data Availability

All data generated or analyzed during this study are included in this published article (and its supplementary information files).
