# Supplementary material for: Hepatocellular Carcinoma cells: activity of Amygdalin and Sorafenib in Targeting AMPK /mTOR and BCL-2 for anti-angiogenesis and apoptosis cell death
Source: BMC Complement Med Ther. 2023 Sep 19;23:329. doi: 10.1186/s12906-023-04142-1 (PMC10508032; doi:10.1186/s12906-023-04142-1)
Supplement: Supplementary file 1 — Supplementary Material 1 [file 12906_2023_4142_MOESM1_ESM.docx]

**Table 1S:** Sequence of primers used in RT-PCR expression analysis

| **Gene** | **Forward primer** | **Reverse primer** | **Accession number** |
| --- | --- | --- | --- |
| **GAPDH** | 5’ TGCACCACCAACTGCTTAGC 3’ | 5’ GGCATGGACTGTGGTCATGAG 3’ | NM_002046 |
| **LC3** | 5’ TTCCGAGTTGCTGACTGACC 3’ | 5’ CCCTTGTAGCGCTCGATGAT 3’ | NM_181509 |
| **Bcl2** | 5’ AGGAAGTGAACATTTCGGTGAC 3’ | 5’ GCTCAGTTCCAGGACCAGGC 3’ | NM_000633 |
| **Beclin 1** | 5’ GGGCTCCCGAGGGATGG 3’ | 5’ GCTGTTGGCACTTTCTGTGG 3’ | NM_001313998 |
| **ATG5** | 5’ GCAACTCTGGATGGGATTGC 3’ | 5’ TTGCAGCAGCGAAGTGTTTC 3’ | NM_004849 |
| **AMPK1** | 5’ ACCAGGTCATCAGTACACCA 3’ | 5’ ATTGTGGCCCTCTTCATGGG 3’ | NM_006251 |
| **mTOR** | 5’ GCCGCGCGAATATTAAAGGA 3’ | 5’ CTGGTTTCCTCATTCCGGCT 3’ | NM_001386500 |
| **HMGB1** | 5’ CATCTCAGGGCCAAACCGAT 3’ | 5’ CCTCTTGGGTGCATTGGGAT 3’ | NM_001313892 |
