# Supplementary material for: Hepatocellular Carcinoma cells: activity of Amygdalin and Sorafenib in Targeting AMPK /mTOR and BCL-2 for anti-angiogenesis and apoptosis cell death
Source: BMC Complement Med Ther. 2023 Sep 19;23:329. doi: 10.1186/s12906-023-04142-1 (PMC10508032; doi:10.1186/s12906-023-04142-1)
Supplement: Supplementary file 2 — Supplementary Material 2 [file 12906_2023_4142_MOESM2_ESM.pdf]

CompuSyn Report

Experiment Name: Amygdalin Sorafenib Combination
Date: 15-11-2020
File Name: C:\Users\hp\Desktop\Combination\Combination Data\HepG2 Combination.cse
Description

Drug: Sorafenib (SF) [umol]
Drug: Amygdalin (AMG) [mg]
Drug Combo: Sorafenib :Amygdalin Combination (SF:AMG) (SF+AMG [1:5])

Data for Drug: SF [umol]

Dose	Effect
8.0	0.42
4.0	0.33
2.0	0.3
1.0	0.26
0.5	0.17

5 data points entered.
X-int: 1.24924
Y-int: -0.5161 +/- 0.02887
m: 0.41311 +/- 0.05536
Dm: 17.7519
r: 0.97410

Data for Drug: AMG [mg]

Dose	Effect
40.0	0.84
20.0	0.8
10.0	0.66
5.0	0.53
2.5	0.44

5 data points entered.
X-int: 0.57364
Y-int: -0.4192 +/- 0.06120
m: 0.73071 +/- 0.05631
Dm: 3.74665
r: 0.99121

Data for Drug Combo: SF:AMG (SF+AMG [1:5])

Dose A	Effect
8.0+	0.99

4.0+	0.98
2.0+	0.97
1.0+	0.69
0.5+	0.6

5 data points entered.

X-int: 0.38802

Y-int: -0.6421 +/- 0.34952

m: 1.65492 +/- 0.30128

Dm: 2.44355

r: 0.95371

Dose-Effect Curve

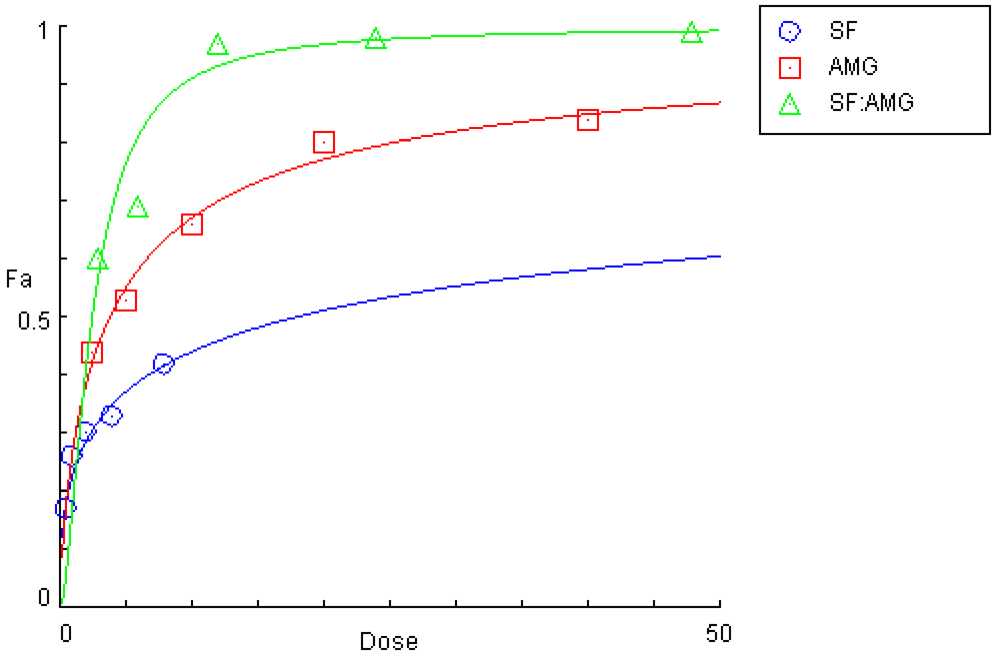

Median-Effect Plot

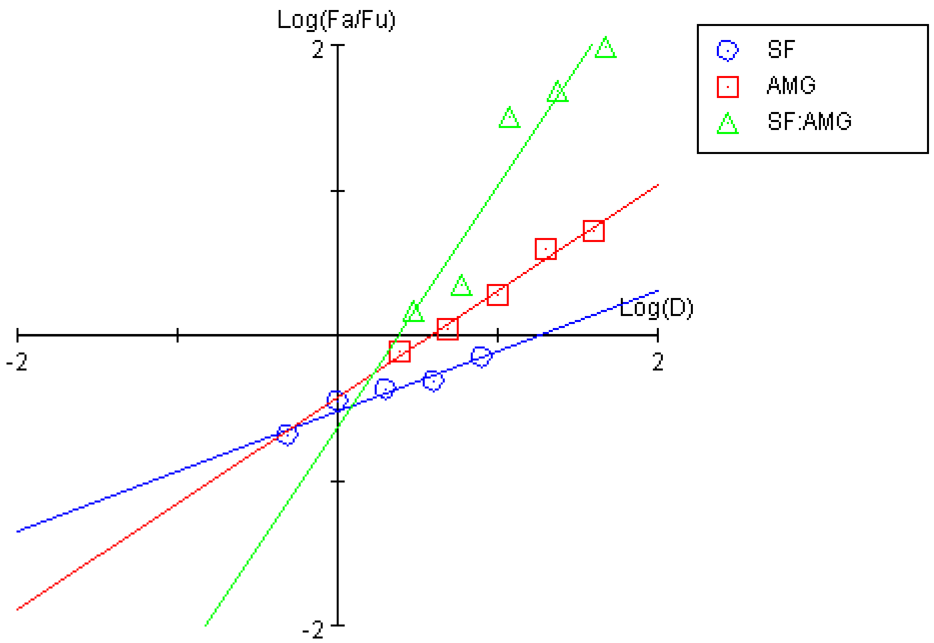

CI Data for Drug Combo: SF:AMG (SF+AMG [1:5])

Fa	CI Value	Total Dose
0.05	9.98132	0.41240
0.1	4.15534	0.64776
0.15	2.58189	0.85667
0.2	1.85251	1.05735
0.25	1.42723	1.25810
0.3	1.14546	1.46443
0.35	0.94292	1.68101
0.4	0.78885	1.91257
0.45	0.66661	2.16451
0.5	0.56644	2.44355
0.55	0.48215	2.75856
0.6	0.40966	3.12195
0.65	0.34609	3.55201
0.7	0.28935	4.07732
0.75	0.23784	4.74599
0.8	0.19024	5.64706
0.85	0.14535	6.96991
0.9	0.10179	9.21790
0.95	0.05737	14.4784
0.97	0.03819	19.9638

CI values for actual experimental points:

Total Dose	Fa	CI Value

48.0	0.99	0.01984
24.0	0.98	0.02598
12.0	0.97	0.02295
6.0	0.69	0.45458
3.0	0.6	0.39366

Combination Index Plot

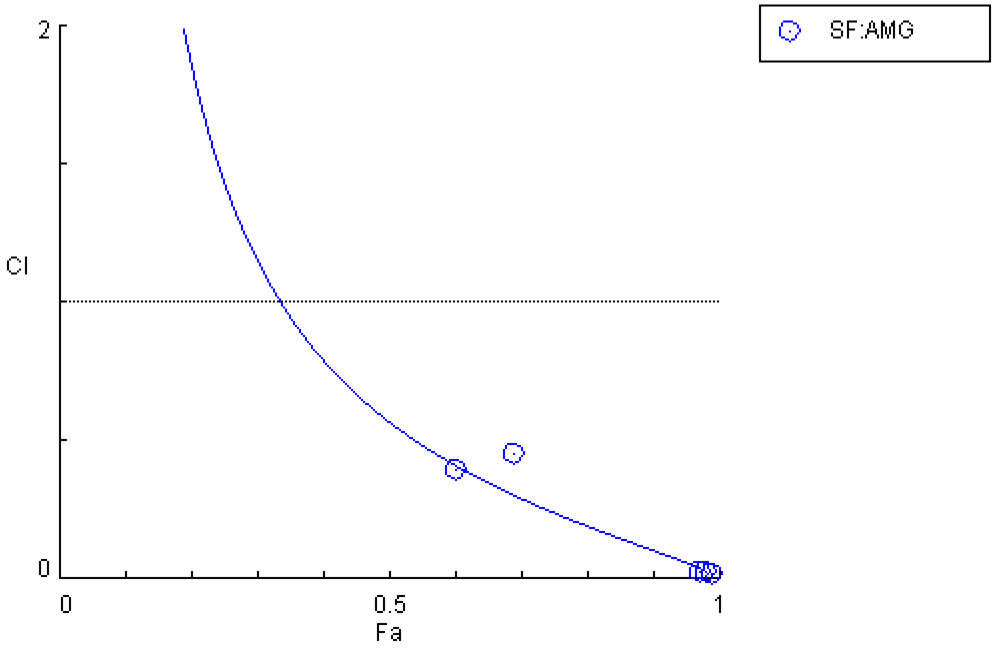

Logarithmic Combination Index Plot

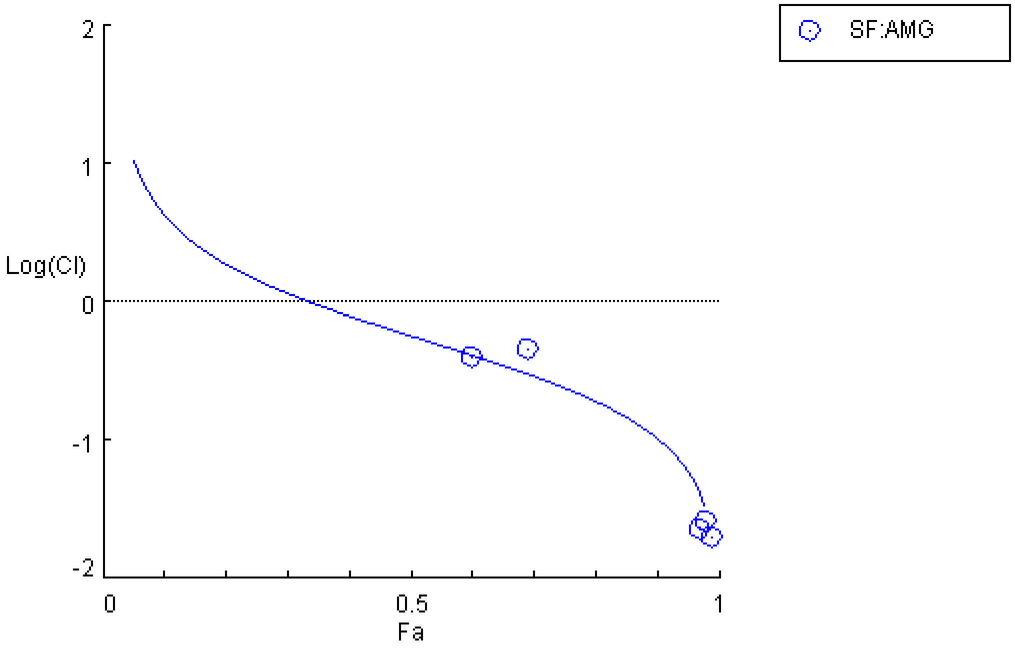

DRI Data for Drug Combo: SF:AMG (SF+AMG [1:5])

Fa	Dose SF	Dose AMG	DRI SF	DRI AMG
0.05	0.01425	0.06663	0.20734	0.19386
0.1	0.08697	0.18524	0.80558	0.34317
0.15	0.26651	0.34890	1.86658	0.48872
0.2	0.61926	0.56197	3.51403	0.63778
0.25	1.24253	0.83309	5.92573	0.79462
0.3	2.28300	1.17506	9.35382	0.96288
0.35	3.96704	1.60591	14.1595	1.14640
0.4	6.65259	2.15109	20.8701	1.34966
0.45	10.9216	2.84693	30.2744	1.57833
0.5	17.7519	3.74665	43.5887	1.83994
0.55	28.8538	4.93072	62.7583	2.14491
0.6	47.3693	6.52571	91.0378	2.50832
0.65	79.4366	8.74106	134.183	2.95305
0.7	138.033	11.9461	203.122	3.51588
0.75	253.618	16.8498	320.631	4.26039
0.8	508.876	24.9790	540.681	5.30805
0.85	1182.43	40.2336	1017.89	6.92697
0.9	3623.41	75.7784	2358.51	9.86495
0.95	22112.7	210.691	9163.72	17.4625
0.97	80086.4	436.148	24069.5	26.2164

DRI values calculated at experimental points

Fa	Dose SF	Dose AMG	DRI SF	DRI AMG
0.99	1202130	2017.04	150266.	50.4261
0.98	219076.	770.397	54768.9	38.5199
0.97	80086.4	436.148	40043.2	43.6148
0.69	123.136	11.1992	123.136	2.23984
0.6	47.3693	6.52571	94.7385	2.61029

DRI Plot for Combo: SF:AMG (SF+AMG [1:5])

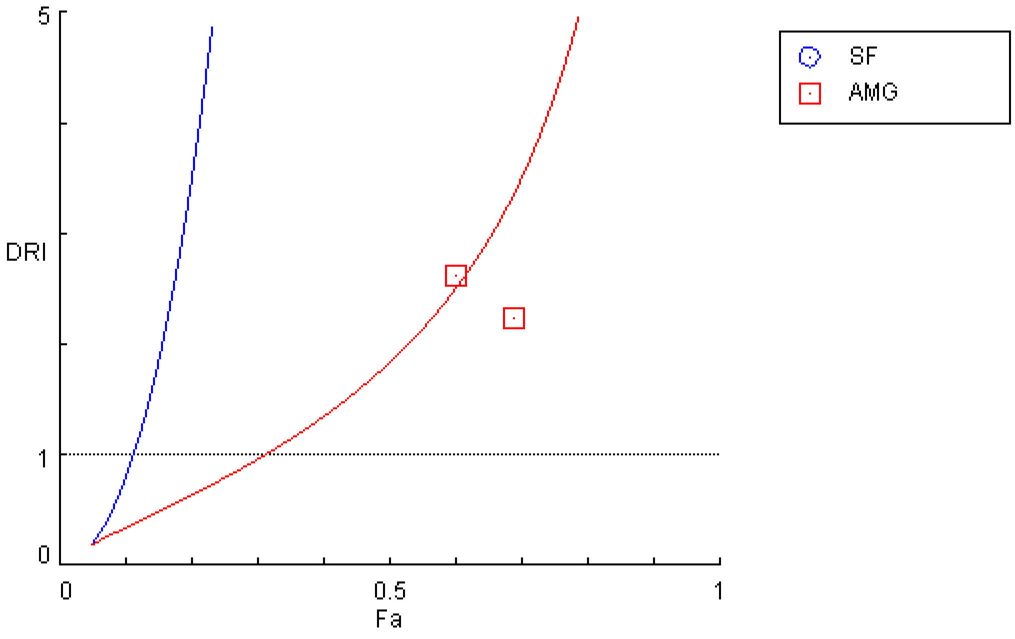

Log(DRI) Plot for Combo: SF:AMG (SF+AMG [1:5])

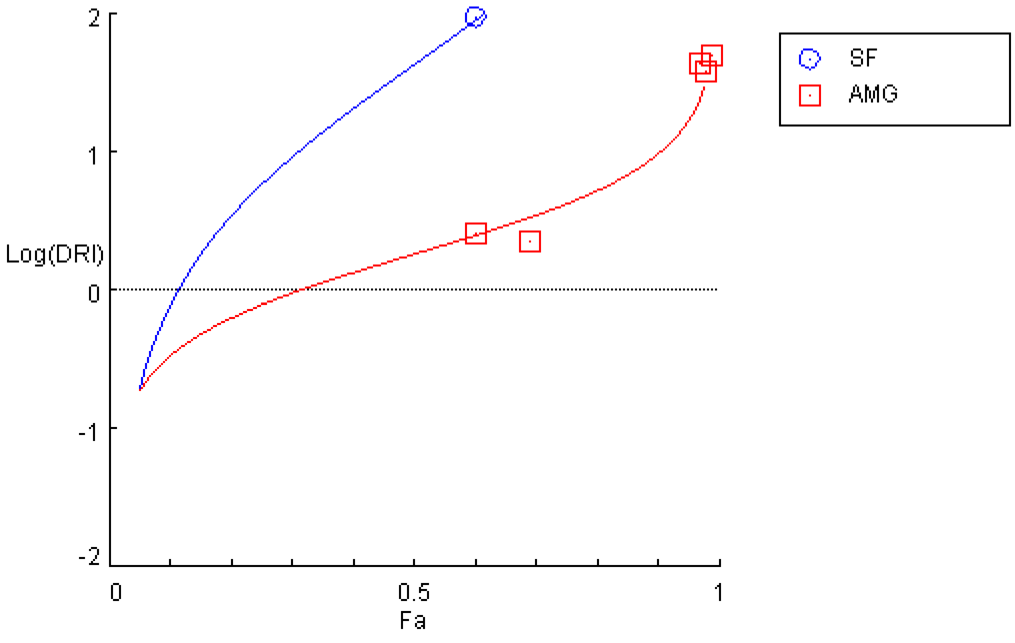

Isobologram for Combo: SF:AMG (SF+AMG [1:5])

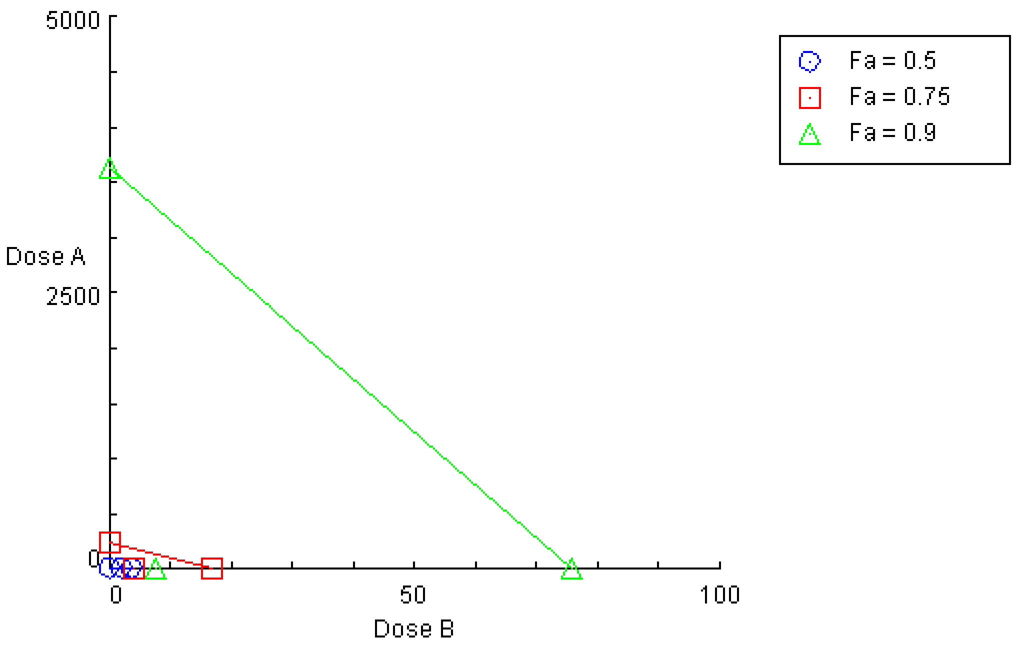

Summary Table

Experiment Name: Amygdalin Sorafenib Combination
Date: 15-11-2020
File Name: C:\Users\hp\Desktop\Combination\Combination Data\HepG2 Combination.cse
Description

Drug: Sorafenib (SF) [umol]
Drug: Amygdalin (AMG) [mg]
Drug Combo: Sorafenib :Amygdalin Combination (SF:AMG) (SF+AMG [1:5])

Drug/Combo	Dm	m	r
SF	17.7519	0.41311	0.97410
AMG	3.74665	0.73071	0.99121
SF:AMG	2.44355	1.65492	0.95371

CI values at:

Combo	ED50	ED75	ED90	ED95
SF:AMG	0.56644	0.23784	0.10179	0.05737

Data for Fa = 0.5

Drug/Combo	CI value	Dose SF	Dose AMG
SF		17.7519	
AMG			3.74665
SF:AMG	0.56644	0.40726	2.03629

Data for Fa = 0.75

Drug/Combo	CI value	Dose SF	Dose AMG
SF		253.618	
AMG			16.8498
SF:AMG	0.23784	0.79100	3.95499

Data for Fa = 0.9

Drug/Combo	CI value	Dose SF	Dose AMG
SF		3623.41	
AMG			75.7784
SF:AMG	0.10179	1.53632	7.68158

Data for Fa = 0.95

Drug/Combo	CI value	Dose SF	Dose AMG
SF		22112.7	
AMG			210.691
SF:AMG	0.05737	2.41307	12.0653

Data for Fa = 0.97

Drug/Combo	CI value	Dose SF	Dose AMG
SF		80086.4	
AMG			436.148
SF:AMG	0.03819	3.32730	16.6365
