# Supplementary figures and images for: Hepatocellular Carcinoma cells: activity of Amygdalin and Sorafenib in Targeting AMPK /mTOR and BCL-2 for anti-angiogenesis and apoptosis cell death
Source: BMC Complement Med Ther. 2023 Sep 19;23:329. doi: 10.1186/s12906-023-04142-1 (PMC10508032; doi:10.1186/s12906-023-04142-1)

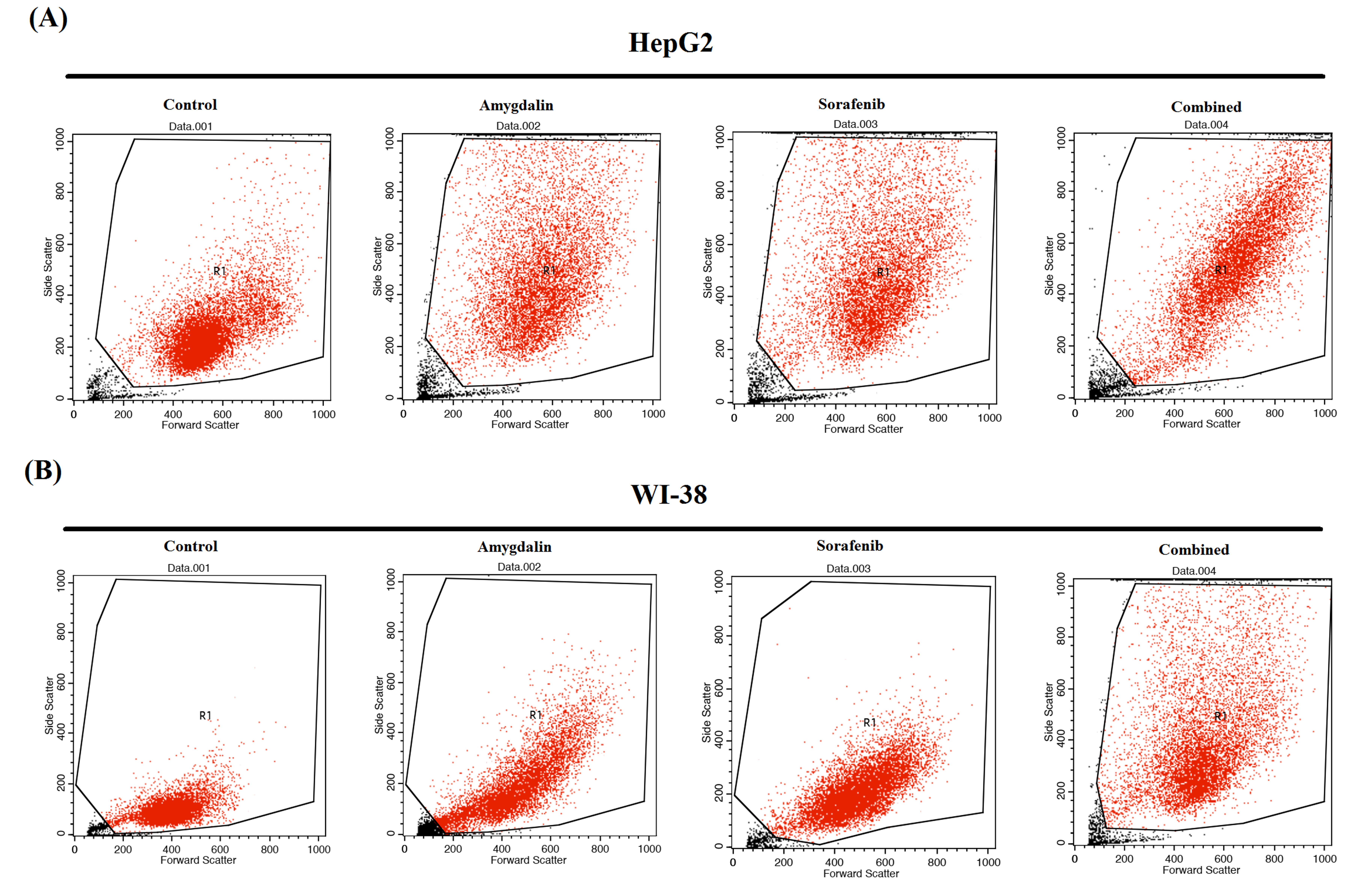

Supplement: Supplementary file 3 — Supplementary Material 3 [file 12906_2023_4142_MOESM3_ESM.tif]

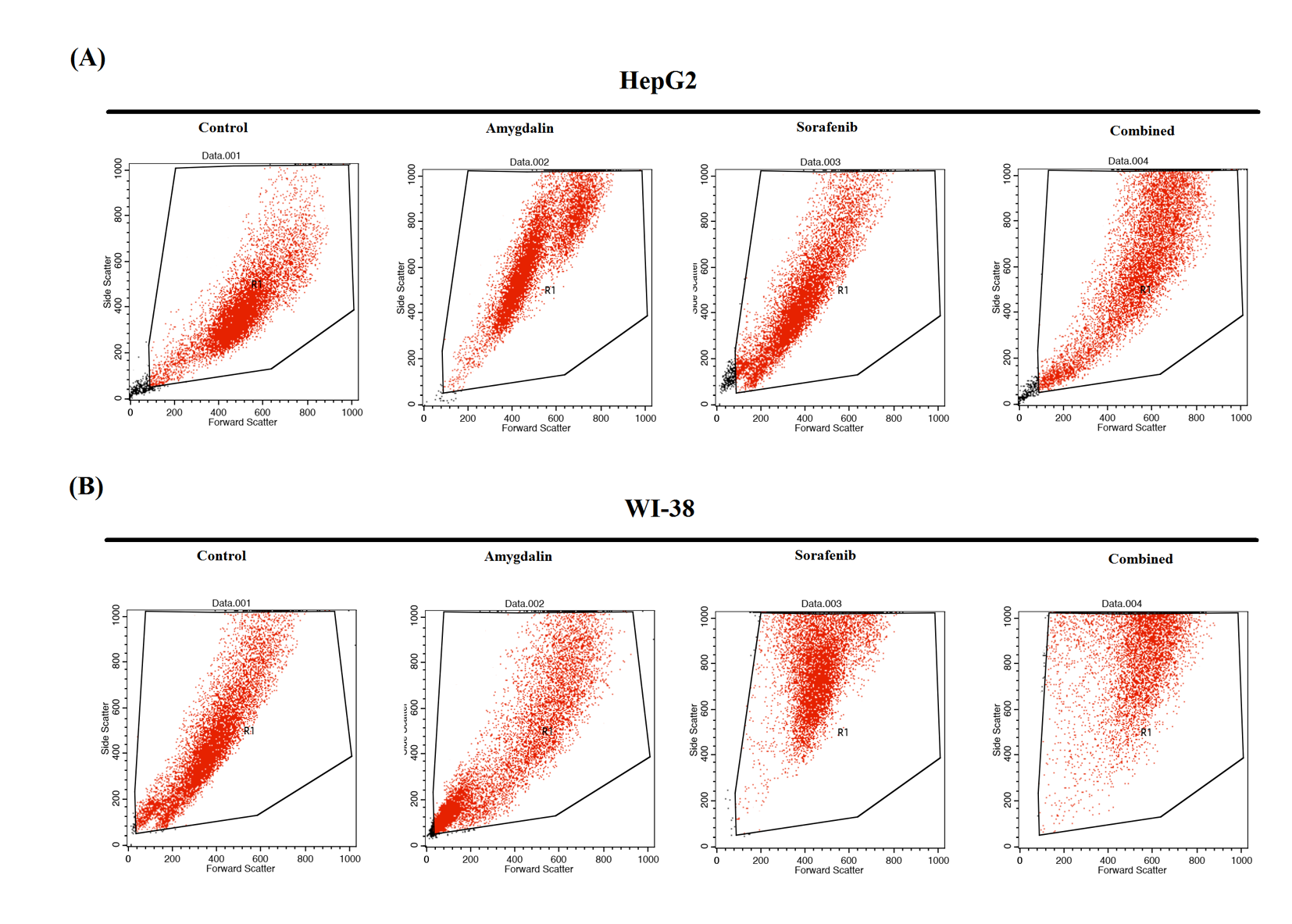

Supplement: Supplementary file 4 — Supplementary Material 4 [file 12906_2023_4142_MOESM4_ESM.tif]
